# Supplementary material for: The causal relationship between the human gut microbiota and pyogenic arthritis: a Mendelian randomization study
Source: Front Cell Infect Microbiol. 2024 Nov 26;14:1452480. doi: 10.3389/fcimb.2024.1452480 (PMC11629706; doi:10.3389/fcimb.2024.1452480)
Supplement: Supplementary file 1 [file DataSheet1.zip › mendelian test/ebi-a-GCST90027708.csv_forest.pdf]

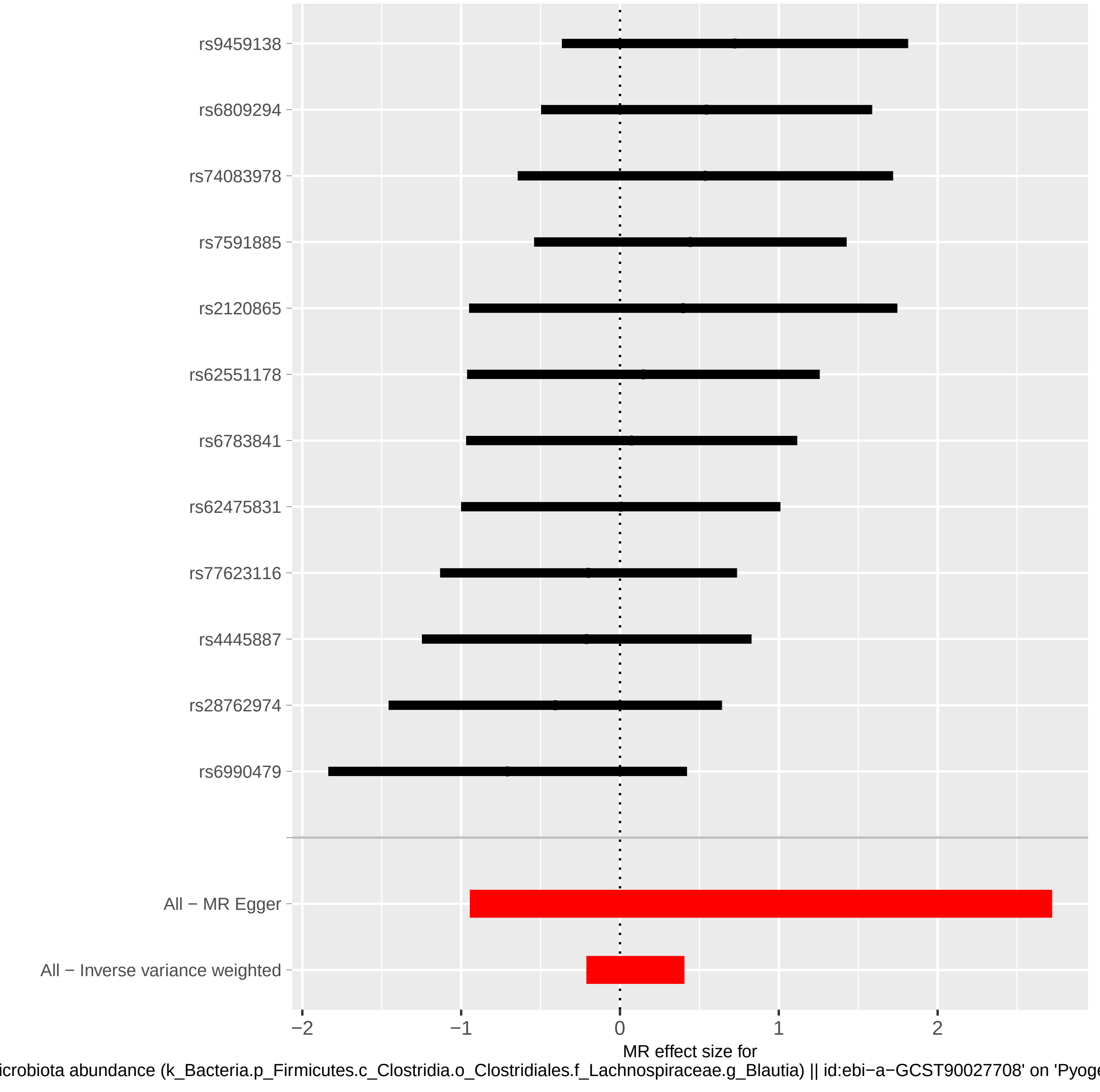

rs9459138
rs6809294
rs74083978
rs7591885
rs2120865
rs62551178
rs6783841
rs62475831
rs77623116
rs4445887
rs28762974
rs6990479
All − MR Egger
All − Inverse variance weighted
−2
−1
0
1
2
MR effect size for
icrobiota abundance (k_Bacteria.p_Firmicutes.c_Clostridia.o_Clostridiales.f_Lachnospiraceae.g_Blautia) || id:ebi−a−GCST90027708' on 'Pyoge
